# Aquaporin-4 in Astroglial Cells in the CNS and Supporting Cells of Sensory Organs—A Comparative Perspective

**DOI:** 10.3390/ijms17091411

**Published:** 2016-08-26

**Authors:** Corinna Gleiser, Andreas Wagner, Petra Fallier-Becker, Hartwig Wolburg, Bernhard Hirt, Andreas F. Mack

**Affiliations:** 1 Institute of Clinical Anatomy and Cell Analysis, Eberhard Karls Universität Tübingen, 72074 Tübingen, Germany; corinna.gleiser@klinikum.uni-tuebingen.de (C.G.); andreas.wagner@anatom.uni-tuebingen.de (A.W.); bernhard.hirt@uni-tuebingen.de (B.H.); 2 Institute of Pathology and Neuropathology, Eberhard Karls Universität Tübingen, 72076 Tubingen, Germany; Petra.Fallier-Becker@med.uni-tuebingen.de (P.F.-B.); h.wolburg@t-online.de (H.W.)

**Keywords:** brain water channel, polarization, astroglial evolution, water homeostasis

## Abstract

The main water channel of the brain, aquaporin-4 (AQP4), is one of the classical water-specific aquaporins. It is expressed in many epithelial tissues in the basolateral membrane domain. It is present in the membranes of supporting cells in most sensory organs in a specifically adapted pattern: in the supporting cells of the olfactory mucosa, AQP4 occurs along the basolateral aspects, in mammalian retinal Müller cells it is highly polarized. In the cochlear epithelium of the inner ear, it is expressed basolaterally in some cells but strictly basally in others. Within the central nervous system, aquaporin-4 (AQP4) is expressed by cells of the astroglial family, more specifically, by astrocytes and ependymal cells. In the mammalian brain, AQP4 is located in high density in the membranes of astrocytic endfeet facing the pial surface and surrounding blood vessels. At these locations, AQP4 plays a role in the maintenance of ionic homeostasis and volume regulation. This highly polarized expression has not been observed in the brain of fish where astroglial cells have long processes and occur mostly as radial glial cells. In the brain of the zebrafish, AQP4 immunoreactivity is found along the radial extent of astroglial cells. This suggests that the polarized expression of AQP4 was not present at all stages of evolution. Thus, a polarized expression of AQP4 as part of a control mechanism for a stable ionic environment and water balanced occurred at several locations in supporting and glial cells during evolution. This initially basolateral membrane localization of AQP4 is shifted to highly polarized expression in astrocytic endfeet in the mammalian brain and serves as a part of the neurovascular unit to efficiently maintain homeostasis.

## 1. Introduction

Aquaporin-4 (AQP4) is a classical, water-specific aquaporin. Although first discovered in the brain [1] AQP4 was subsequently localized in many other organs [2,3]. Playing a role in fast water transport through epithelia seems to be its prevalent function in the body. This includes epithelia in the lung, salivary glands, intestinal tract, to name just a few [4,5,6] (see also other articles in this issue). So far, the reports on its cellular distribution showed its presence exclusively in the basolateral membrane domain whereas other aquaporins have also been localized to the apical membrane domain. Besides epithelial cell expression, AQP4 occurs also on skeletal muscle.

In the vertebrate central nervous system (CNS), AQP4 is expressed by astrocytes or astroglial cells, and ependymal cells. Astrocytes are one of the two main types of macroglial cells in the vertebrate CNS, ependymal cells are lining the brain ventricles and are sometimes considered a separate class of glial cells However, they share some features with astrocytes, one of them is the expression of AQP4. In sensory epithelia, AQP4 has been found in supporting cells.

Up to about 30 years ago, glial cells were considered glue or structural support cells (“Nervenkitt”) which had little to do with information processing [7]. When astrocytes were recognized as being more than structural support, their enzymatic activity and ion channels were studied and it was established that they are necessary for transmitter recycling, nutritional support, and potassium removal from the extracellular space (trophic, metabolic, and synaptic functions) [8,9].

**Figure 1:**
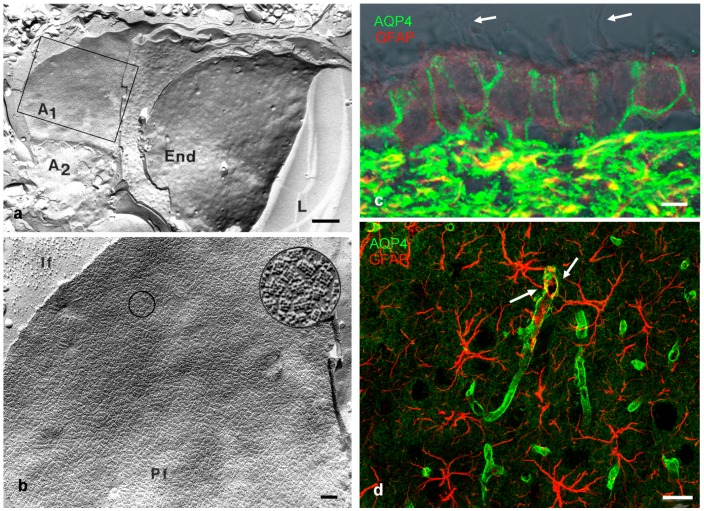
(**a**) Freeze fracture replica of rat brain tissue surrounding a capillary: the fracture plane goes through the lumen (L) of a capillary and the bordering endothelial cell (End). This is surrounded by astrocytes A1 and A2. The membrane of A1 indicated by the square is shown at higher magnification in b; (**b**) High densities of orthogonal arrays of particles (OAP) are found on astrocytic endfeet facing a basal lamina. The circled area is magnified further showing OAPs on the P-face (Pf) If: intermediate filaments; (**c**) Ependymal cells lining the ventricle; and (**d**), hippocampal astrocytes in rat brain stained for glial fibrillary acidic protein (GFAP, **red**) and AQP4 (**green**). Ependymal cells are positive for AQP4 at their basolateral membranes, bearing cilia apically (arrows). Brain astrocytes in d show high AQP4 binding at their endfeet surrounding capillaries (arrows). Scale bars: (**a**) 1 µm; (**b**) 0.1 µm; (**c**) 5 µm; (**d**) 20 µm; (**a**,**b**) are modified from [24].

The history of the discovery of aquaporins in the mammalian brain is curiously remarkable in the respect that the main water channel in the CNS, aquaporin-4 (AQP4), was investigated on ultra-morphological grounds before the first reports on the existence of specific water channels were published. So-called orthogonal arrays of particles (OAPs) were found in membranes of astrocytes by the freeze fracture method employing electron microscopy [10,11]. These OAPs occurred especially in high densities at places where astrocytes faced the basal lamina and therefore extracellular fluid spaces as is the case around blood vessels (Figure 1a,b) and under the pia mater, at the superficial glia limitans [12]. Before it became known that OAPs consist of AQP4, there were many speculations on their functional involvement ranging from ion channels [13] to regeneration, and even a role for water transport, reviewed in Wolburg [12]. The suggestion for a role in regeneration resulted from comparisons of various brain structures across different species, reviewed in [14]. Within the brain, OAPs were also found in membranes of ependymal cells, the cells that line the ventricular surface [15], and of course in many cells outside the CNS [16].

At this time, the involvement of OAPs in water transport was speculated on just by few researchers [17] and not accepted in the research community, nor was there good evidence for it. In 1986, Benga and co-workers published evidence for the existence of such a channel [18], and in 1992, the first water channel was cloned by the Agre group [19], and soon after water channels were found in the CNS [1], and as the forth water channel sequenced it was named aquaporin-4 (AQP4). For the history of the discovery of water channels, see Benga [20]. The first indication that OAPs were formed by water channels originated from studies of AQP4 knock-out mice that did not form any OAPs [21]. Direct evidence in support of the idea that the particle arrays consisted of water channel proteins was then provided by freeze fracture immunolabeling [22]. This notion is now generally accepted.

Since then, aquaporins in the vertebrate brain have been suggested to be involved in many physiological processes such as waste removal and fine-tuning of potassium homeostasis, reviewed in [23] some of which we will discuss below.

In this review, we focus on AQP4 as the main water channel occurring in the vertebrate brain, and its expression in the brain and in neuro-sensory structures, more specifically the retina, inner ear, olfactory mucosa, and taste buds. In addition, we will include data on non-mammalian AQP4 expression to arrive at an evolutionary perspective.

## 2. Structure and Localization of Aquaporin-4 (AQP4)

Like other aquaporins, AQP4 occurs as tetramers [25] with two major isoforms, M1 and M23 differing in their initiation sites at Met1 or Met23, respectively at the N terminus [1], reviewed in Papadopoulos [26]. The combined expression of both isoforms determines the formation of arrays as multiples of heterotetramers [27,28,29]. When CHO-K1 cells were transfected with M1 isoform of AQP4, no arrays formed in the membranes, whereas transfecting with the M23 isoforms led to large raft-like lattices. The expression of both isoforms after transfection resulted in OAPs that resembled those of astrocytes in vivo [30], suggesting that both isoforms coexist in one array [31]. With the development of superresolution techniques in light microscopy, some attempts have been undertaken to study array formation by localization microscopy: Transfecting cells with fluorescent-tagged AQP4 M1 and M23 isoforms showed that the longer M1 isoform was more mobile in the cell membrane than the shorter M23, suggesting a stronger interaction at the N-terminus of the latter [32]. Two-color dSTORM indicated that M1 and M23 co-assemble in OAPs with a M1-enriched periphery surrounding a M23-enriched core [33]. Further studies suggested that M1-M23 aggregation state has effects on the AQP4 mobility in the plasma membrane [29]. Although many reports have scrutinized the expression patterns and conditions of square array and higher order structures of AQP4, the precise function of array formation remains elusive [16]. Suggestions range from ideal high density packaging to adhesion function, gas transport, and anchoring at the basement membrane, summarized in [23].

AQP4 has been described to be expressed in epithelial cells of many organs, including kidney, intestine, salivary glands, sensory organs, and skeletal muscle [3]. In all cases of epithelial cell expression, AQP4 was found to be localized in the basolateral membrane domain [14,34]. This general principle, however, is not followed in the specialized epithelium of the cochlea, nor in the neuroepithelium derived CNS. As will be outlined and discussed below, two of inner ear supporting cell types express AQP4 exclusively in the basal membrane domains, and CNS astrocytes show high AQP4 presence at their endfeet membranes.

## 3. AQP4 Distribution in the (Mammalian) Brain

In the mammalian brain, antibody studies as well as freeze fracture EM revealed a high density of AQP4 expression on astrocytic endfeet membranes at the perivascular and the superficial glia limitans (Figure 1c,d). This expression pattern of AQP4, and other channel expression such as the potassium channel Kir4.1, indicates that astrocytes are highly polarized cells. This is not surprising in the sense that epithelial cells are polarized cells, and astrocytes are derived from neuroepithelial cells during development [35]. Yet, due to their star-shaped morphology, there are no defined apical and basolateral membrane domains in astrocytes, nor is there a sub-apical tight junction. The perivascular endfeet are part of the neurovascular unit and directly behind the actual blood-brain barrier (BBB) formed by the endothelial cell tight junctions [36].

Several aspects may shed light on the function of AQP4 distribution in the mammalian brain. Firstly, AQP4 expression does not start in a polarized pattern of cellular distribution, but is spread over cell membrane of radial glial cells and early astrocytes during cortical development [37]. The beginning of polarized expression coincides roughly with the appearance of OAPs. Several studies have established that the microenvironment and molecules of the extracellular matrix such as the dystrophin associated protein complex and agrin play a decisive role in AQP4 polarization and OAP formation [35,38,39,40,41,42]. Secondly, in ependymal cells AQP4 is expressed in basolateral membrane domains (Figure 1c) but not in the specialized ependymal cells covering the stroma and blood vessels of the choroid plexus. These latter cells form subapical tight junctions (the basis for the blood-cerebrospinal fluid barrier) and express aquaporin-1 in the apical membrane but no AQP4 [14,16]. This is similar to circumventricular organs where the blood-brain barrier is shifted from the endothelial to the glial barrier. Thirdly, in response to pathological conditions, such as traumatic injury, stroke, or tumor, astrocytes are reactive and often upregulate intermediate filaments, and also AQP4. This increase correlates with a loss in polarized AQP4 expression and a reduction in OAPs [43].

## 4. AQP4 Expression in Sensory Structures

### 4.1. Olfactory Epithelium

**Figure 2:**
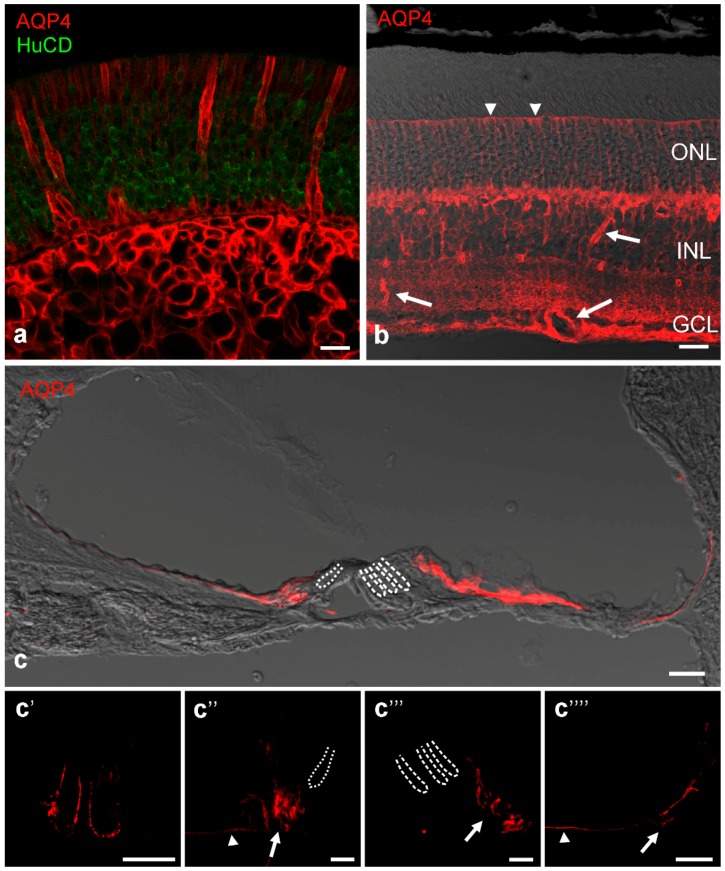
AQP4 immunocytochemical stains (**red**) in rodent sensory organs. (**a**) Rat olfactory region shows supporting epithelal cells and cells of Bowman’s glands positively stained. Receptor cells labelled by antibodies against the RNA-binding proteins HuC/D as a neuronal marker (**green**) are negative for AQP4; (**b**) AQP4 in the mouse retina is especially strong around blood vessels (arrows) but immunoreactivity is also present along the Müller cell processes and even at the outer limiting membrane (arrow heads). ONL: outer nuclear layer, INL: inner nuclear layer, GCL: ganglion cell layer; (**c**) AQP4 immunolabeling in the rat cochlear duct: In the medial part of the cochlear duct AQP4 was localized in the inner sulcus cells, and in the lateral part of the cochlear duct in Hensen’s cells and Claudius cells. The supporting cells of the organ of Corti were devoid of labeling for AQP4. The interdental cells and outer sulcus cells were out of focal plane. In **c′**–**c′′′′** detailed confocal images of the subcellular AQP4 expression in the rat cochlear duct are shown: (**c**′) In the medial part of the cochlear duct AQP4 is localized in the basolateral membranes of the interdental cells; (**c**′′) the basal membrane domains of the inner sulcus cells (arrow head), and the basolateral membrane domains of the inner sulcus cells directly adjacent the organ of Corti (arrow); (**c**′′′) In the lateral part of the cochlear duct AQP4 is localized in the basolateral membrane domains of Hensen’s cells (arrow); and (**c**′′′′) the basal membranes of Claudius cells (arrow head). The outer sulcus cells with their root process show AQP4 expression in the basolateral membrane domains (arrow). Dotted lines represent the localization of inner hair cells, dashed lines mark outer hair cells. Scale bars represent 20 µm in all images.

In the sensory epithelium of the olfactory mucosa, supporting cells surrounding the receptor cells are clearly positive for AQP4 in the basolateral membrane domain [44,45] (Figure 2a). This is also true for nasal mucosa cells in the non-sensory regions and cells of the Bowman glands. Receptor cells as well as ensheathing glia wrapping the olfactory fila do not express AQP4. The expression of AQP4 on supporting cells and mucosal epithelium cells was confirmed by the detection of OAPs [45]. Functionally, AQP4-null mice showed significant impairments in smell tests [46]. Thus, AQP4 water channels likely provide an osmotic environment that facilitates the appropriate binding of odorant molecules to receptor and binding proteins.

### 4.2. Retina

The retina is derived from the diencephalon and therefore part of the central nervous system. Accordingly, Müller cells, the radial glia specific to the retina show an AQP4 distribution in a pattern similar to CNS astrocytes: AQP4 expression is high at locations where Müller cell processes face a basal lamina like around blood vessels and at the inner limiting membrane [47] where high densities of OAPs also occur [48,49]. There, Müller cells form endfeet that express AQP4 often but not always co-localized with the inward rectifying potassium channel Kir 4.1 [50]. Together, these channels are thought to be involved in regulating water homeostasis in the retina [51]. Other non-endfoot membranes of Müller cells are also moderately positive for AQP4, including the region of the outer limiting membrane but not the microvilli (Figure 2b) [47]. This is reflected in the report that Müller cells show lower densities of the scaffold proteins dystrophin and a-syntrophin known to be involved in AQP4 polarization compared to cortical astrocytes [52]. In addition to Müller cells, astrocytes occur in retinas with a superficial vascular plexus in the nerve fiber layer [53,54]. Interestingly, the blood vessels surrounded by Müller cell processes showed blood-retinal barrier impairment in AQP4-KO mice, but not the vessels covered by astrocytic processes, which displayed a stronger gliotic response [54]. In Müller cell endfeet, AQP4 co-localized with the transient receptor potential cation channel TRPV4; AQP4 and TRPV4 have been reported to synergistically interact in cell volume regulation and influence each other in their gene expression [55]. Increased AQP4 expression has been observed in animal models of induced diabetes [56], and AQP4 knockdown in diabetic animals led to aggravation of retinopathy [57].

### 4.3. Inner Ear

Within the mammalian inner ear, the expression of AQP4 in the epithelial supporting cells of the cochlea is acquired at the onset of hearing function [58]. The neuro-sensory epithelium of the cochlea consists of different supporting cell types exhibiting a distinct AQP4 expression pattern, reviewed in Eckhard et al. [59]: In most supporting cell types of the cochlear duct, AQP4 is expressed in the basolateral membrane domains (Figure 2c), however two distinct supporting cell types (inner sulcus cells and Claudius cells) showed expression of AQP4 only in the basal membrane. In the supporting cells of the organ of Corti, directly flanking the sensory cells, AQP4 was not found [59,60,61]. Whether these differences in AQP4 polarity and expression arise due to morphological constraints or have a functional role is unknown. The functional significance of AQP4 expression in the cochlear duct is clearly noticeable in AQP4-deficient mice showing a pan-cochlear moderate hearing impairment [62,63]. The importance of AQP4 for normal hearing has been further supported by a mutation screening, in which a single nucleotide polymorphism could be found in the AQP4 gene of one deaf patient [64]. All these studies suggested that AQP4 is involved in maintaining the osmotic balance in the supporting cell epithelium that recycles K^+^ from the side of sensory transduction in the organ of Corti back to the current generator in the stria vascularis [61,62,63]. As in Müller cells of the retina [50] and astrocytes of the CNS [65,66], co-localization of Kir4.1 and AQP4 in the supporting cells of the cochlear duct was found, enabling a rapid volume-equilibration in response to osmotic challenge [59]. However, the lack of AQP4 just in the cochlear supporting cells at the transduction sites of the K^+^ recycling routes [59] is in contrast to the functional coupling of AQP4 and K_ir_4.1 described for the CNS [65,66] and for the retina [50]. Moreover, in contrast to the glial cells in CNS and retina, the distribution of AQP4 does not completely coincide with the distribution of OAPs in the supporting cells of the cochlea. The Claudius cells in the cochlear duct, exhibit a strictly basal expression of AQP4 and a concomitant lack of OAPs, whereas supporting cells showing a basolateral AQP4 expression also exhibit OAPs (Figure 2c). Further, the supporting cells of the cochlea, particular the Hensen’s cells show an inter- and intracellular heterogeneity in the distribution, size and shape of OAPs, never observed in other tissues under non-pathological conditions [61].

### 4.4. Taste Buds

There have been no reports to our knowledge on the expression on AQP4 in the cells of taste buds, and our own staining experiments were negative. However, other aquaporins (AQP1, AQP2, and AQP5) were localized to rat taste buds by immunocytochemistry [67].

### 4.5. Somatosensory Ganglia

The cell bodies of somatosensory neurons are located in ganglia in the dorsal root of the spinal nerves connecting to the spinal cord, and in trigeminal ganglia. The expression of AQP4 in these ganglia has been demonstrated by immunocytochemistry, Western blot, and PCR analysis [68]. Immunoreactivity was restricted exclusively to satellite cells, a type of glial cell surrounding neuronal pericarya. No other cell type, especially no Schwann cell, expressed AQP4. This is constistent with the much earlier finding of OAPs on satellite cells in these ganglia [69]. No particular membrane distribution of AQP4 was observed, and no specific function suggested as AQP4-lacking mice showed no sensory deficits [68].

### 4.6. AQP4 in Sensory Cells

In the CNS, AQP4 is restricted to glial cells, neurons do not express this fast water channel. The same is true for sensory cells or neurons, at least in mammalian sensory organs. However, data have been presented that imply the expression of AQP4 in hair cells in the amphibian papilla of the leopard frog (*Rana pipiens*) [70]. AQP4 immunoreactivity localized primarily to the apical and basal poles of hair cells but supporting cells were AQP4-negative. Another study reported AQP4 immunoreactivity in sensory organs of the zebrafish (*Danio rerio*) but the low power micrographs do not allow to distinguish between sensory and supporting/glial cells [71]. In the retina of gilthead sea bream (*Sparus aurata*) a marine teleost fish, AQP4 was localized to Müller cells like in mammals but in addition to one type of amacrine cell, and cone photoreceptors [72]. With this limited evidence, further studies are needed to elucidate the scope of AQP4 expression in sensory cells.

## 5. Glia and AQP4 Distribution in the Brain of Fish and Other Non-Mammalian Vertebrates

The two major macroglial cells in the CNS, astroglial and oligodendroglial cells, are present in most vertebrates, including teleost fish. Unlike in mammals however, the astroglial cells in fish do not show the typical star-shaped morphology; they rather have long processes and often contact the ventricle and the pial surface of the brain [73,74]. They are referred to as radial glial cells, present for example in the optic tectum and can at the same time be considered ependymal cells since they contact the ventricle, and no other specialized cuboidal cells lining the ventricle exist like in the mammalian brain [75]. These adult radial glial cells form junctional complexes at the ventricular surface [74] and thus retain epithelial-like polarity. According to the infolding of the neural tube during development, the side facing the ventricle represents the apical side, the pial side, the basal or basolateral surface.

**Figure 3:**
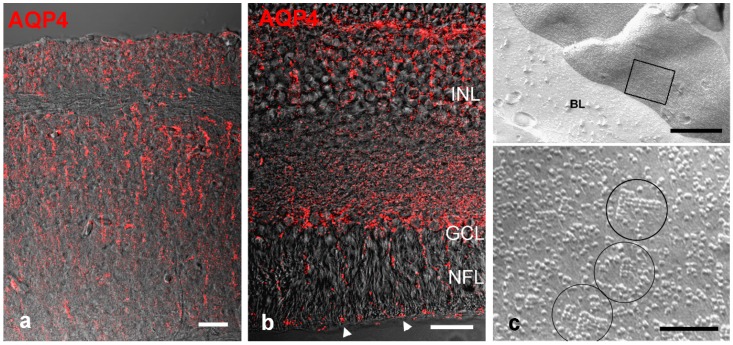
AQP4 localization fish brain and retina. (**a**) Immunostain in the brain (optic tectum) of a zebrafish (*Danio rerio*). AQP4 is expressed along radial glial fibers, but not particularly enhanced at the brain surface nor around blood vessels; (**b**) In the retina of the cichlid fish *Astatotilapia burtoni* punctate stain for AQP4 is detected along Müller cell fibers and endfeet at the inner limiting membrane (arrow heads); (**c**) Freeze fracture electron micrograph through Müller cell endfeet facing a basal lamina (BL). The square area shown at higher magnification in the lower panel reveals OAPs (circled). INL: inner nuclear layer, GCL: ganglion cell layer, NFL: nerve fiber layer. Scale bars 20 µm in (**a**,**b**), and 0.5 µm and 0.1 µm in (**c**).

When stained for AQP4 by immunohistochemistry, radial glial cells in the zebrafish brain were positive but did not show the polarized distribution as it is known from mammalian astrocytes [74]. OAPs occurring in high density in mammalian perivascular astrocytic endfeet have never been found in the brain of fish. Thus, the expression pattern of AQP4 on radial glial cells and the lack of OAPs in fish brain resemble the situation of radial glial cells during cortical development. However, the radial glial (Müller) cells of the goldfish (*Carassius auratus*) retina have been reported to form OAPs at their endfeet facing the vitreous humor [76], a finding we could recently confirm in the retina of cichlid fish *Astatotilapia burtoni* (Figure 3). Thus, the formation of OAPs by AQP4 on glial cells is a trait that evolved prior to tetrapod evolution but is not prevalent in the fish brain.

In amphibians and reptiles, radial glial types predominate, although smaller astrocyte-like cells with multiple processes might have evolved several times during vertebrate evolution. Aquaporins were present at the beginning of deuterostome and vertebrate evolution. This includes AQP4 as one of the classical aquaporins [77]. Besides in the teleost CNS, the expression of AQP4 in the brain of sharks has been documented [78]. However, information on the distribution of AQP4 in the brain of other fish groups, reptiles, and amphibians is scarce. Freeze-fracture data from the 1980–1990s (summarized in [16]) revealed OAPs on retinal Müller cells in all major vertebrate groups. In amphibians, Müller cells of urodeles formed OAPs, whereas those of anurans did not. In the lizard thoracic spinal cord OAPs were present, but the caudal spinal cord was OAP-negative. Generally, the brains of elasmobranchs, hagfish, or lamprey are completely devoid of OAPs.

In birds, astrocytes are formed and show more or less the mammalian pattern of AQP4 distribution in brain [79,80] and retina [81].

## 6. Aquaporin and the Evolution of Central Nervous System (CNS) Structure

All major groups of eukaryotic organisms show expression of water channels [82]. Within vertebrates, aquaporin 4 has been reported to occur in the gills of the jawless hagfish [83] confirming much earlier reports on OAPs [84], and in many tissues of sharks including kidney, gill, and brain [85]. In the gills of hagfish, there was clearly basolateral expression of AQP4, but a polarized expression on astroglial processes as seen in mammals has not been demonstrated. It is noteworthy that a glial-based BBB was common in early vertebrate brain evolution [86].

Although many functions have been suggested for AQP4 besides water transport such as facilitating cell migration and cell adhesion, the control of water balance and homeostasis is likely its main role. In the evolutionary context, it is interesting that in the sarcopterygean lineage leading to tetrapods, an ancient aquaporin gene cluster evolved and diverged into paralogous forms of AQP2, -5, or -6 [77]. This presumably enabled increased water conservation necessary for survival in terrestrial habitats. In the actinopterygian lineage, a genome duplication presumably occurred during early teleost evolution. Thus, 18 members of the aquaporin gene family were identified in the zebrafish [87], more than in mammals where 13 AQPs are usually found (numbered AQP0-12) [82]. Regarding AQP4, two *AQP4* gene sequences have been predicted for the cichlid fish *Astatotilapia burtoni*, and we have recently confirmed the expression of both genes in brain and retina of this fish (unpublished observations). For further aspects on the evolution of aquaporin genes and their occurrence in vertebrate phylogeny, see [77].

The high density of AQP4 around blood vessels in the mammalian brain is thought to facilitate water flow when potassium is released from the endfeet to extracellular space and blood vessels (potassium siphoning) thus serving ionic homeostasis [88,89]. In addition, a convective water flow from perivascular arterial spaces through nervous system parenchyma has been suggested to play a major role in waste removal [90,91]. This is, in part, a paracellular fluid movement but is thought to be facilitated by AQP4 channels and their polarized distribution. Impairment of AQP4 leads to accumulation of waste products in the brain, which might be one of causes for neurodegenerative diseases. Re-distribution of AQP4 in brain diseases such as tumors can lead to cytotoxic edema. Thus, the polarized distribution in the mammalian brain is essential for water balance and ionic equilibration. As pointed out above, in fish brain, the shape and AQP4 distribution of astroglial cells differs from the situation in mammalian astrocytes. Since there is a lack or low degree of polarized distribution of water channels, the fluid movements in the fish brain are likely different from the mammalian brain. This is corroborated by the maintained radial morphology of astroglial cells in the fish brain suggesting an apical-basal (i.e., surface-ventricular) dominated polarity rather than a perivascular-parenchymal polarity. Thus, a bulk convective water flow as suggested for the mammalian brain seems unlikely in the fish brain.

A strict polarity with a basolateral AQP4 expression is maintained in the supporting cells of the sensory epithelia of the olfactory mucosa. A more differentiated expression is seen in the inner ear where inner sulcus cells and Claudius cells show a restricted basal expression. This AQP4 expression is likely providing a water balance to control together with ion channels the ionic concentration surrounding the sensory cells, thereby enabling them to transduce sensory stimuli into voltage changes. Interestingly, in the mammalian retina, the AQP4 polarization on radial glial processes around blood vessels and expression of scaffolding proteins is less pronounced than in astrocytes. In mammalian astrocytes, there is a strong polarity reflected in AQP4 expression but a basolateral domain cannot be defined. An overview of the AQP4 expression patterns is presented in Table 1.

Derouiche et al. [92] proposed three membrane domains for Müller cells and astrocytes, ventricular, neuronal, and basal lamina facing, based on distinct molecule localizations including AQP4. Given the heterogeneity of astrocytes in the mammalian brain [93], even more differentiated domain subdivisions might be anticipated.

In summary, a polarized expression of AQP4 is part of a control mechanism for a stable ionic environment. This is beneficial for the neighboring sensory or nerve cells and neuronal signaling. This is easily achieved in epithelial-like structures of sensory organs, i.e., the basolateral membrane domain of supporting cells. In the mammalian brain, this polarity is shifted to astrocytic endfeet at the endothelial and superficial glia limitans and serves as a part of the neurovascular unit to efficiently maintain homeostasis.

## Figures and Tables

**Table 1:** Cellular membrane distribution of AQP4.

Sensory Structure or CNS Cells	Basolateral Membrane	Basal Membrane Domain	Highly Polarized	OAPs
Olfactory Supporting Cells	+			+
Supporting Cells Organ of Corti	+	+		+
		Inner sulcus and Claudius cells		(not all cells)
Teleost Fish Retinal Müller Cells	+	+		+
Mammalian and Avian Retinal Müller Cells			+	+
Teleost Fish Brain Radial Glia	+			
Mammalian Ependymal Cells	+			+
Mammalian and Avian Astrocytes			++	+

+ positive; ++ strongly positive.
